# Preliminary results demonstrating the impact of Mediterranean diet on bone health

**DOI:** 10.1186/s12967-017-1184-x

**Published:** 2017-04-24

**Authors:** Maria Cristina Savanelli, Luigi Barrea, Paolo Emidio Macchia, Silvia Savastano, Andrea Falco, Andrea Renzullo, Elisabetta Scarano, Immacolata Cristina Nettore, Annamaria Colao, Carolina Di Somma

**Affiliations:** 1 I.O.S. & COLEMAN Srl, Acerra, 80011 Naples Italy; 2 Dipartimento di Medicina Clinica e Chirurgia, Unit of Endocrinology, Federico II University Medical School of Napoli, Via Sergio Pansini 5, 80131 Naples, Italy; 3 IRCCS SDN, Napoli Via Gianturco 113, 80143 Naples, Italy

**Keywords:** Bone health, Mediterranean diet, Environmental factors, Imaging techniques, Calcaneal quantitative ultrasound (QUS) scanner

## Abstract

**Background:**

Nutrition is an environmental factor affecting bone health. Nutrition is considered essential to achieve and maintain optimal bone mass. Mediterranean diet (MD) has shown to prevent bone disease. Aim of this study is to investigate the relationship between bone health status and adherence the MD.

**Methods:**

Four-hundred eighteen healthy people (105 males and 313 females, age 50 ± 14 years) were recruited in the outdoor hospital of the “Campus Salute Onlus” held in Piazza del Plebiscito in Naples, October 17–20th 2013 and 09–11th October 2014. All subjects underwent clinical assessment, calcaneal quantitative ultrasound (QUS) scanner and PREvención con DIeta MEDiterránea (PREDIMED) questionnaire.

**Results:**

Globally, prevalence of osteoporosis and osteopenia were 7.7 and 46.0%, respectively. The majority of subjects (60.5%) had an average score (score 6–9) of adherence to MD. The T-score showed positive correlation with PREDIMED score (r = 0.250, *p* < 0.001). The higher T-scores were positively associated with a higher consumption of extra-virgin olive oil (EVOO), vegetables, fruits, legumes, and fish and negatively associated with consumption of red meat. The higher T-scores were positively associated with the highest odds of PREDIMED scores (higher adherence) (OR 6.91, IC 6.27–7.61, *p* < 0.001). Multiple regression analysis models indicated that, among the single food items investigated, high T-score can be predicted by consumption of EVOO (*p* < 0.001), fish (*p* < 0.001) and fruit (*p* = 0.002) intake. A PREDIMED score of 3 was found to be predictive for a low T-score (α = 0.05, R-squared index = 0.417).

**Conclusions:**

The results demonstrate a positive correlation between bone health status and adherence to MD, suggesting that a high adherence to MD promotes bone health. The observations here reported confirmed that a specific dietary approach, such as MD, can represent a modifiable environmental factor for osteoporosis’ prevention.

## Background

Osteoporosis is a systemic skeletal disease characterized by low bone mass and microarchitectural deterioration of bone tissue with a consequent increase in bone fragility and increased risk of susceptibility to fracture [1]. Osteoporosis prevalence rises with age and, in consideration of the demographic transition occurring worldwide, it is projected to significantly increase in the next future. In Italy, approximately 3.5 million persons are osteoporotic, and more than 90,000 fractures are yearly reported in subjects older than 50 years [2]. Mortality associated with osteoporotic fractures ranges from 15 to 30%, with a rate similar to breast cancer and stroke [2]. Indeed, osteoporosis and osteoporosis-related fractures are associated with high morbidity and mortality, reduced quality of life and increase in healthcare costs [2]. Therefore, effective prevention and treatment strategies are necessary to prevent osteoporosis and to reduce the risk of osteoporosis-related fractures.

Prevention and management of osteoporosis require suitable methods for population screenings and early diagnosis. Currently, dual-energy X-ray absorptiometry (DXA), with its excellent predictive value for fracture risk, is considered the gold standard imaging technique for the diagnosis of osteoporosis [2]. According to the World Health Organization (WHO), osteoporosis is defined as a bone mineral density (BMD) at the hip and/or the spine at least 2.5 standard deviations below the mean peak bone mass of young healthy adults as determined by DXA [3]. The use of DXA as method for population screenings and primary care diagnosis presents several critical limitations, including the use of ionizing radiation, the large size of the equipment, its high costs and its limited availability. Therefore, in the last years it is grown the interest for alternative and reliable pre-screening devices for osteoporosis assessment, such as the quantitative ultrasound (QUS) scanners [4–6]. Compared to DXA, QUS offers wider accessibility to the public since the apparatus is portable, easier to handle, has a lower cost and not emits ionizing radiation [4–6]. Studies in humans have demonstrated that QUS can reflect bone quality and predict risk for future fracture, and therefore QUS can be used as tool for population screening of osteoporosis [4–6].

Several risk factors have been identified for primary osteoporosis [1, 2]. Among these, nutrition is widely accepted as an environmental factor affecting bone health. Adequate nutrition is essential to achieve and maintain optimal bone mass and to prevent osteoporosis [7, 8]. Many nutritional factors have been suggested to play a potential role in the risk modulation of this disabling disease. Historically, calcium and vitamin D were the primary nutrients considered for osteoporosis prevention, however nutritional benefits for bone have been demonstrated for several additional dietary factors, including vitamins (A, B, C, E, K), minerals (potassium, magnesium, silicon) and macronutrients (protein and fats). All these factors may influence bone and mineral homeostasis and modulate the long-term bone health [7, 8]. In this context, Mediterranean diet (MD) is a specific eating pattern that combines several foods and nutrients proposed to act as protective factors in the development and progression of several chronic diseases [9].

To date, the relationships between bone health and MD adherence have been evaluated only in a limited number of cross-sectional studies, with contrasting results. Therefore, the aim of the present study is to investigate the relationship between bone status, assessed by calcaneal QUS, and the degree of adherence to the MD in healthy subjects.

## Subjects and methods

### Population study

Four-hundred eighteen healthy people (105 males and 313 females, medium age 50 ± 14 years) were recruited as volunteers in the outdoor hospital of the “Campus Salute Onlus” held in Piazza del Plebiscito in Naples, October 17–20th 2013 and 09–11th October 2014 [10].

These subjects were included within the project to investigate the role of lifestyle in preventing chronic diseases supported by the Campus Salute association of Campania. The study is part of a large database started in 2010 to investigate the health status of the general population of Campania Region as analyzed by the free consultation, visit, and diagnostics for people coming to the outdoor hospital held in different public squares of our Region [10].

The work has been carried out in accordance with the Code of Ethics of the World Medical Association (Declaration of Helsinki), and it has been approved by the Ethical Committee of the University of Naples “Federico II” Medical School. The purpose of the protocol was explained to all enrolled subjects, and written informed consent was obtained.

### Clinical assessment

To avoid any inter-operator variability, all anthropometric measurements were recorded by the same operator (a well-trained nutritionist) in standard way, with subjects wearing only light clothes and without shoes. Weight and height were measured to calculate the body mass index (BMI) [weight (kg) divided by height squared (m^2^), kg/m^2^]. Height was measured to the nearest 1 cm using a wall-mounted stadiometer (Seca 711; Seca, Hamburg, Germany). Body weight was determined to the nearest 50 g using a calibrated balance beam scale (Seca 711; Seca, Hamburg, Germany). BMI was classified according to WHO’s criteria with normal weight: 18.5–24.9 kg/m^2^; overweight, 25.0–29.9 kg/m^2^; grade I obesity, 30.0–34.9 kg/m^2^; grade II obesity, 35.0–39.9 kg/m^2^; grade III obesity ≥40.0 kg/m^2^. Waist circumference (WC) was measured to the nearest 0.1 cm with a non-stretchable measuring tape.

According to the National Cholesterol Education Program’s Adult Treatment Panel III (NCEP-ATP III) criteria, abdominal obesity was defined as WC ≥ 102 cm in men and ≥88 cm in women [11]. Hip circumference (HC) was measured as the maximum circumference around the buttocks posteriorly and the symphysis pubis anteriorly, and measured to the nearest 0.5 cm. Waist-to-hip ratio (WHR) was calculated as the waist divided by the HC.

In all subjects, information on smoking habit and physical activity were recorded with a standard questionnaire. Current smokers were defined as those who smoked at least one cigarette per day and former smokers as those who had stopped smoking more than 1 year before the interview; the rest of the participants were defined as noncurrent smokers. Physical activity level was expressed according to whether the participant habitually engaged at least 30 min/day of aerobic exercise (YES/NO). Data of subjects referring any concomitant diseases have been removed from the analysis.

### Bone health status assessment: calcaneal quantitative ultrasound (QUS)

In all subjects, QUS of the mid-calcaneus was performed by the same well-trained operator with a Sahara Clinical Sonometer (Hologic, Bedford, MA, USA). Calibration of the apparatus was performed daily, and measurement were recorded according to the manufacturer’s instructions. Briefly, measurements were performed with the patient seated and its foot positioned and secured in the Sahara system with a positioning aide. After the patient’s foot was secured, a pair of soft elastomer pads were brought into contact with opposite sides of the patient’s heel with a motorized caliper mechanism. Each of the elastomer pads were acoustically coupled to the heel and to the sound transducer using Sahara Ultrasound Coupling Gel. Inaudible high frequency sound waves, produced by one of the sound transducers, were transmitted through the heel and received by the opposite transducer. Quantitative parameters describing the speed and attenuation of the sound waves in the heel were measured. Ultrasound devices routinely measure two parameters: broadband ultrasound attenuation (BUA) and speed of sound (SOS) and the combination of these two measured values allow to calculate the quantitative ultrasound index (QUI), sometimes referred as “stiffness” in the scientific literature. The QUI/Stiffness value obtained for the patient is then converted into estimated BMD (in units of g/cm^2^) and T-score. According to the WHO, patients were considered osteopenic when T-score was −1/−2.5 and were considered osteoporotic when T-score was lower than −2.5 [3]. Reproducibility of the apparatus was 3% C.V. for Estimated Heel BMD; 2.6% C.V. for QUI; 0.22% C.V. for SOS and 3.7% C.V. for BUA.

### Adherence to the Mediterranean diet: PREDIMED questionnaire

Adherence to the MD was measured using a validated 14-item questionnaire (PREDIMED) [12]. The questionnaire was recorded for all the enrolled subjects during a face-to-face interview between the patient and a certified nutritionist or an endocrinologist. Briefly, for each item was assigned score 1 and 0; PREDIMED score was calculated as follows: score 0–5, low adherence; score 6–9, average adherence; score ≥ 10, high adherence [12].

### Statistical analysis

Results are expressed as mean ± SD or as median plus range according to variable distributions evaluated by Kolmogorov–Smirnov test (*p* < 0.01). The correlations between study variables were performed using Pearson *r* or Spearman’s *rho* correlation coefficients. Bivariate proportional odds ratio (OR) models were performed to assess the association among quantitative variables and qualitative variables (sex and each item included in PREDIMED questionnaire). In these analyses, we entered only those variables that had a *p* value <0.05 in the univariate analysis (partial correlation). Using T-score as dependent variables, a multiple linear regression analysis models (stepwise method) was set up to estimate the predictive value of each item included in PREDIMED questionnaire [expressed as r^2^, Beta (β) and t]. To avoid multicollinearity, variables with a variance inflation factor (VIP) > 10 were excluded. Values ≤5% were considered statistically significant. Data were stored and analyzed using the MedCalc^®^ package (Version 12.3.0 1993–2012 MedCalc Software bvba-MedCalc Software, Mariakerke, Belgium). Proportional odds model was carried out using the R Project for Statistical Computing 2014 (http://www.R-project.org).

## Results

The studied population consisted in 418 subjects (25.1% men). The prevalence of current smoking was 17.5%, and physical activity was practiced by 31.8% of the enrolled subjects. Anthropometric and bone health status assessment are summarized in Table 1. The mean BMI was 26.7 ± 3.1 kg/m^2^ for men and 27.1 ± 4.6 kg/m^2^ for women. According to BMI, the majority of subjects was in the overweight range. Values of WC higher that the optimal cut offs were found in 36.2% of men and in 57.5% of women. Globally, prevalences of osteoporosis and osteopenia were 7.7 and 46.0%, respectively. In Table 2 are shown the prevalences of osteoporosis and osteopenia according to gender. There were no differences in bone health status between men and women.

**Table 1:** Anthropometric measurement and bone health status assessment

Parameters	Patients n = 418
Age (years)	50.2 ± 14.0
Anthropometric variables	
Weight (kg)	71.0 (46.0–147.0)
Height (m)	1.6 (1.4–1.9)
BMI (kg/m^2^)	26.4 (18.2–39.9)
Normal weight n (%)	147 (35.2%)
Overweight n (%)	181 (43.3%)
Obesity grade I n (%)	66 (15.8%)
Obesity grade II n (%)	24 (5.7%)
WC (cm) men	98.6 ± 10.0
WC (cm) women	90.7 ± 12.5
HC (cm) men	102.0 (80.0–135.0)
HC (cm) women	102.0 (68.0–147.0)
WHR men	1.0 (0.8–1.1)
WHR women	0.9 (0.6–1.2)
Bone health status assessment	
T-score	−1.0 (−3.6–3.6)
Qui/Stiff	87.3 ± 19.8
BMD	0.5 ± 0.1

The mean BMI was 26.7 ± 3.1 kg/m^2^ for men and 27.1 ± 4.6 kg/m^2^ for women. According to BMI, the majority of subjects was in the overweight range. Values of WC higher that the optimal cut offs were found in 36.2% of men and in 57.5% of women

BMI, body mass index; WC, waist circumference; HC, hip circumference; WHR, waist-to-hip ratio; QUI/Stiff, quantitative ultrasound index/stiffness; BMD, bone mineral density

**Table 2:** Prevalence of osteoporosis and osteopenia according to gender

Parameters	All patients n, % 418	Men n, % 105	Women n, % 313	*χ* ^*2*^	*p* value
Normal	193, 46.3	51, 49.0	142, 45.4	0.21	0.648
Osteopenia	192, 46.0	50, 48.1	142, 45.4	0.08	0.774
Osteoporosis	33, 7.7	4, 2.9	29, 9.2	2.51	0.113

In all subjects, prevalences of osteoporosis and osteopenia were 7.7 and 46.0%, respectively

**Fig. 1:**
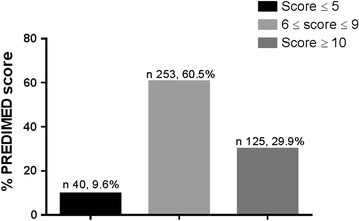
Total PREDIMED score. Score 0–5, lowest adherence to the Mediterranean diet (MD); score 6–9, average adherence to the MD; score ≥ 10, highest adherence to the MD. *PREDIMED,* PREvención con DIeta MEDiterránea

All participants to the study completed the PREDIMED questionnaires. Response frequency of dietary components included in the PREDIMED questionnaire of the subjects are reported in Table 3. Vegetables were the most consumed food item, followed by fruit. The mean PREDIMED score in the subjects was 8.4 ± 2.2. A higher percentage of subjects (60.5%) had an average adherence score (score 6–9) as assessed by the PREDIMED questionnaire. The total PREDIMED score is reported in Fig. 1.

**Table 3:** Response frequency of dietary components included in the PREDIMED questionnaire of the subjects

	Questions	n	%
1	Use of extra virgin olive oil as main culinary lipid	322	77.0
2	Extra virgin olive oil >4 tablespoons	281	67.2
3	Vegetables ≥2 servings/day	398	95.2
4	Fruits ≥3 servings/day	370	88.5
5	Red/processed meats <1/day	112	26.8
6	Butter, cream, margarine <1/day	149	35.6
7	Soda drinks <1/day	256	61.2
8	Wine glasses ≥7/week	181	43.3
9	Legumes ≥3/week	185	44.3
10	Fish/seafood ≥3/week	246	58.9
11	Commercial sweets and confectionery ≤2/week	223	53.3
12	Tree nuts ≥3/week	197	47.1
13	Poultry more than red meats	211	50.5
14	Use of sofrito sauce ≥2/week	363	86.8

### Correlation studies

**Fig. 2:**
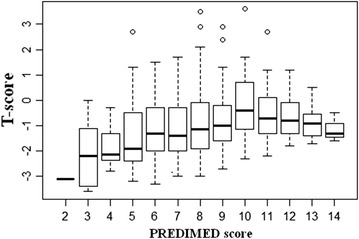
Values of the PREDIMED score predicting the lower T-score. PREDIMED score 3 (α = 0.05, R-squared index = 0.417) was found to be predictive for a lower T-score. The box plot regression shows how the categories under 8 include patients with a critical T-score (−2.5); increasing T-score tends towards range and not include values below −2.5

The T-score showed positive correlation with PREDIMED score (r = 0.250, p < 0.001). After adjustment for BMI, this associations remained significant (r = 0.272, *p* < 0.001). A bivariate proportional odds ratio model was performed to assess the association between T-score and food items of PREDIMED questionnaire (Table 4). In the studied population, higher T-scores were positively associated with a higher consumption of some of the Mediterranean food items, in particular extra-virgin olive oil (EVOO), vegetables, fruits, legumes, and fish. In contrast, the higher T-scores were negatively associated with consumption of red meat (Table 4). The multinomial logistic regression of T-score with PREDIMED score are reported in Table 5. The higher T-scores were positively associated with the higher odds of PREDIMED score (OR 6.91, IC 6.27–7.61, *p* < 0.001). The multiple regression analysis models indicate that, among the single food items of MD, T-score was well predicted by use of EVOO, fish and fruits intake (Table 6). In addition, a PREDIMED score of 3 was found to be predictive for a lower T-score (α = 0.05, R-squared index = 0.417) (Fig. 2).

**Table 4:** Bivariate proportional odds ratio model to assess the association between T-score and food items included in the PREDIMED questionnaire

	Questions	Simple correlation			After adjuster for sex, age and BMI		
		OR	*p* value	95% IC	OR	*p* value	95% IC
1	Use of EVOO as main culinary lipid		*<0.001*	−0.099–0.220		*<0.001*	−0.047–0.298
	Yes	3.59			3.62		
	No	0.75			0.74		
2	EVOO >4 tablespoons		*0.003*	0.096–0.461		*0.006*	0.079–0.458
	Yes	2.06			2.08		
	No	1.31			1.30		
3	Vegetables ≥2 servings/day		*0.012*	0.131–1.044		*0.048*	0.035–1.014
	Yes	1.75			1.63		
	No	1.54			1.66		
4	Fruits ≥3 servings/day		*<0.001*	0.598–1.312		*<0.001*	0.507–1.250
	Yes	2.55			2.36		
	No	1.06			1.15		
5	Red/processed meats <1/day		*0.048*	−0.383–0.004		0.139	−0.347–0.044
	Yes	0.82			0.86		
	No	3.28			3.15		
6	Butter, cream, margarine <1/day		0.329	−0.083–0.248		0.326	−0.087–0.261
	Yes	1.08			1.09		
	No	2.50			2.49		
7	Soda drinks <1/day		0.732	−0.134–0.194		0.062	−0.005–0.359
	Yes	1.02			1.19		
	No	2.64			2.28		
8	Wine glasses ≥7/week		0.570	−0.209–0.114		0.648	−0.131–0.209
	Yes	0.95			1.04		
	No	2.84			2.68		
9	Legumes ≥3/week		*0.019*	0.033–0.359		*0.028*	0.020–0.360
	Yes	2.23			1.20		
	No	1.21			2.25		
10	Fish/seafood ≥3/week		*<0.001*	0.529–0.949		*<0.001*	0.540–0.976
	Yes	2.08			2.12		
	No	1.30			1.28		
11	Sweets and confectionery ≤2/week		0.594	−0.203–0.116		0.870	−0.180–0.153
	Yes	0.95			0.98		
	No	2.83			2.76		
12	Tree nuts ≥3/week		0.179	−0.273–0.049		0.974	−0.208–0.214
	Yes	0.89			1		
	No	3.03			2.70		
13	Poultry more than red meats		0.462	−0.099–0.220		0.157	−0.047–0.298
	Yes	1.06			1.13		
	No	2.56			2.40		
14	Use of sofrito sauce ≥2/week		0.173	−0.069–0.429		0.103	−0.037–0.495
	Yes	1.18			1.24		
	No	2.28			2.18		

In the studied population, higher T-scores were positively associated with a higher consumption of some of the Mediterranean food items, in particular extra-virgin olive oil (EVOO), vegetables, fruits, legumes, and fish and were negatively associated with consumption of red meat

EVOO, extra-virgin olive oil; BMI, body mass index

*p* value in italic emphasis denotes a significant difference (p < 0.05)

**Table 5:** Multinomial logistic regression of T-score with PREDIMED score

PREDIMED score	OR	*p* value	95% IC
Low adherence	3.25	*<0.001*	2.95–3.58
Average adherence	6.80	*<0.001*	6.17–7.49
Higher adherence	6.91	*<0.001*	6.27–7.61

The higher T-score were positively associated with the higher odds of PREDIMED score

*PREDIMED,* PREvención con DIeta MEDiterránea

*p* value in italic emphasis denotes a significant difference (p < 0.05)

**Table 6:** Multiple regression analysis models (stepwise method) with the T-score as dependent variable to estimate the predictive value of food items of PREDIMED questionnaire

Parameters	Multiple Regression analysis			
Model 1	R^2^	*β*	t	*p* value
EVOO	0.173	0.419	9.41	*<0.001*
Fish	0.225	0.244	5.36	*<0.001*
Fruit	0.241	0.140	3.13	*0.002*

At multiple regression analysis models, among adherence to the MD and single food items, T-score was well predicted by use EVOO, fish and fruit intake

Variable excluded, other food items of PREDIMED questionnaire

*EVOO,* extra-virgin olive oil

*p* value in italic emphasis denotes a significant difference (p < 0.05)

## Discussion

In this work, the relationship between bone health status and adherence to MD have been investigated in 418 healthy people. Volunteers were recruited during a project to study the role of lifestyle in the prevention of chronic diseases supported by the Campus Salute association of Campania, held in Piazza del Plebiscito in Naples, October 17–20th 2013 and 09–11th October 2014 [10].

In the studied population, prevalence of osteoporosis and osteopenia (measured using calcaneal QUS) were 7.7 and 46.0% respectively. The majority of subjects (60.5%) had a PREDIMED score corresponding to an average adherence to MD. The interesting finding of our study was that T-score was positively associated with PREDIMED score; particularly, at the multinomial logistic regression of T-scores with PREDIMED score, the higher T-scores were positively associated with the highest odds of PREDIMED score. This finding suggest that a higher adherence to MD is associated with better bone health status.

To date, it is widely accepted that adequate nutrition is essential to achieve and maintain optimal bone mass, and dietary approaches can represent an important strategy for osteoporosis prevention [7, 8]. Several nutritional factors are known to play a role in skeletal health. Among these, calcium and vitamin D are considered the primary nutrients for osteoporosis prevention in older adults [7, 8]. However, emerging evidences indicate that additional nutrients such as vitamins (A, B, C, E, K), minerals (potassium, magnesium, silicon) and macronutrients (protein and fats) may contribute to skeletal health by supporting both bone matrix production and mineralization [7, 8].

Recent evidences reported differences in the severity of osteoporosis across the European Union Countries, suggesting a lower incidence of the disease in the Mediterranean area. This effect has been mainly attributed to specific eating pattern [13, 14]. The term “Mediterranean Diet” is currently used in the literature to indicate the traditionally dietary habits of the people living in countries bordering the Mediterranean Sea. Indeed, MD consists in a high consumption of EVOO, vegetables, legumes, whole grains, fruits and nuts, a moderate consumption of poultry and fish (varying with proximity to the sea), a low consumption of dairy products and red meat, and low-to-moderate consumption of wine as the main source of alcohol accompanying meals [9]. The protective role of MD against several diseases, such as cardiovascular, neoplastic, neurodegenerative and other chronic diseases is now well defined [9, 15–19]. One of the most accredited hypothesis for this association suggests that the protective effects of MD are mediated by the anti-inflammatory properties of beneficial compounds, like polyphenols, that are largely present in the Mediterranean foods such as vegetables, fruits and red wine [20]. In particular, EVOO is one of components of the MD and represent the main edible fat. Its increased consumption is reflected in the high monounsaturated to saturated fatty acid and has been found to be associated with a reduced prevalence of risk factors for major chronic inflammatory diseases included osteoporosis [7, 13, 21]. Adherence to MD has been associated with higher BMD and has been shown to prevent bone disease [9, 13, 14, 22–24]. The Framingham Osteoporosis Study Cohorts previously showed that a dietary pattern similar to MD was predictive of greater BMD in adults [22]. Similarly, adherence to the MD, measured as a Mediterranean diet score, was confirmed to have beneficial effects on BMD in healthy women from Southern Spain [23]. The positive correlation between bone health parameters and adherence to the MD was also identified in a previous report of our group, on a small sample of elderly (>65 years) [24]. In addition, the potential protective effect of the MD against the risk of hip fractures has been also studied and a higher adherence to MD was associated with a lower risk in both genders [25–27]. In contrast, different studies suggested that a high consumption of fish and EVOO and a low red meat intake rather than MD per se were positively correlated with bone mass [28–30].

The positive effects of MD on bone could be attributed to the combined beneficial effects of individual components of the MD. Accordingly, the present study reported the specific association between T-score and food items included in the PREDIMED questionnaire. A higher T-score was significantly associated with a higher consumption of EVOO, vegetables, fruits, legumes and fish and negatively associated with consumption of red meat. Among the specific food items included in MD, EVOO, fish and fruits are the best predictors of T-score.

Of note, in the present work adherence to MD has been evaluated using the 14-items questionnaire of the PREDIMED study [12]. This is less time-demanding, less expensive and requires less collaboration from participants than the usual full-length food frequency questionnaire (FFQ) or other more comprehensive methods [20]. Moreover, this questionnaire allows to provide feedback to the participant immediately after the interview is completed. Nevertheless, this method is very reliable, being previously validated against the FFQ used in the study [31].

In this study, the bone status has been evaluated using the calcaneal QUS. We are aware that DXA is the most commonly used and validated method for bone densitometry in clinical practice [2], however it should be considered that the study has been performed in an outdoor hospital held in a public squares. In these conditions, QUS is more suitable for a population screening, since the apparatus is portable and easier to handle. Moreover, QUS has been confirmed to be able to predict risk for future fracture in humans [4–6]. In particular, the calcaneus has been chosen as a site for measurement since it is easily accessible, well-suited for optimizing the geometry of transmission of the ultrasound (US) wave through it, and it contains approximately 90% trabecular bone which present a high metabolic turnover rate and a pattern of bone loss similar to the spine [6]. For these reasons, QUS is becoming a frequently used method for the assessment of bone quality even if it can only be considered as a pre-screening tool. A subsequent confirmatory diagnosis is necessary with a DXA evaluation [4–6].

An additional limit of the present study is that information concerning vitamin D status, calcium intake and body composition of the enrolled subjects are missing. Indeed, in the present study we could obtain preliminary information on the bone health status of healthy subjects and correlate the results with a specific dietary pattern.

## Conclusions

In summary, this work demonstrates a positive correlation between bone health status and adherence to MD. The results suggest that higher adherence to MD plays a beneficial role in bone health and confirm that a specific dietary approach, such as MD, can represent an important modifiable environmental factor for osteoporosis’ prevention.

We are aware that the results reported here are preliminary. Large-scale studies are required to clarify the real effect of MD and of its individual components on bone health and on osteoporosis’ prevention, as well as the validity of QUS as preliminary method for the diagnosis of this disease, when DXA is unsuitable for population screenings.

## Abbreviations

DXA: dual-energy X-ray absorptiometry
BMD: bone mineral density
QUS: quantitative ultrasound scanner
MD: Mediterranean diet
PREDIMED: PREvención con DIeta MEDiterránea
BMI: body mass index
WC: waist circumference
HC: hip circumference
WHR: waist-to-hip ratio
EVOO: extra-virgin olive oil
